# Preparation of Thin Film Composite (TFC) Membrane with DESPs Interlayer and Its Forward Osmosis (FO) Performance for Organic Solvent Recovery

**DOI:** 10.3390/membranes13070688

**Published:** 2023-07-24

**Authors:** Jingyi Liang, Hansheng Huang, Hao Zhang, Yanhui Wu, Yongbing Zhuang

**Affiliations:** 1 School of Chemical Science and Engineering, Tongji University, Shanghai 200092, China; 2 Shanghai Key Laboratory of Chemical Assessment and Sustainability, Tongji University, Shanghai 200092, China; 3 Institute of Process Engineering, Chinese Academy of Sciences, Beijing 100190, China

**Keywords:** organic solvent forward osmosis, composite membrane, interlayer, ethanol, monascorubrin

## Abstract

To explore the application of forward osmosis (FO) technology in the organic solvent recovery field, we prepared a new solvent-resistant triple layer thin film composite (TFC) membrane on the PI (polyimide) substrate. The deep eutectic supramolecular polymers (DESPs) interlayer was constructed on the substrate to improve the separation performance and solvent resistance. DESPs interlayer was formed by mixing and heating with cyclodextrin as the hydrogen bond acceptor and *L*-malic acid as the hydrogen bond donor. The chemical changes, surface property and morphology of the composite membrane with DESPs interlayer were characterized. The separation performance and stability of the triple layer composite membrane in organic solvent FO were studied. For the monascorubrin-ethanol system, the permeation flux of TFC/DESPs5-PI membrane could reach 9.51 LMH while the rejection rate of monascorubrin was 98.4% (1.0 M LiCl/ethanol as draw solution), which was better than the pristine membrane. Therefore, this solvent-resistant triple layer composite FO membrane has good potential for the recovery of organic solvents.

## 1. Introduction

Organic solvents are widely used in the petrochemical, pharmaceutical, food, cosmetics, and fine chemical industries [1,2,3]. Most organic solvents have toxicity and do great harm to the environment and human health. Therefore, the removal, recovery and reuse of the waste organic solvents in the industrial processes are essential to improve the sustainable development [4]. The traditional method used for organic solvent recycling is distillation, which is a process of high-energy consumption [5,6]. With the advances in separation techniques, adsorption and membrane separation have also been applied in the field of organic solvent treatment [7,8]. Recently, organic solvent nanofiltration (OSN) has been widely studied. Compared with distillation, it shows advantages of no phase change and low energy consumption. However, the high-operating pressure and fouling tendency may increase the cost [9,10]. Forward osmosis (FO), a novel membrane process, has the advantages of low energy consumption and low pollution. And recently, a new concept of organic solvent forward osmosis (OSFO) has been proposed [9]. In 2018, Chung et al. [10] used the OSFO process for simultaneous concentration of pharmaceutical products and recovery of organic solvents, and demonstrated that the OSFO process is a promising technology for solvent recovery as it possesses a reasonable solvent flux, low reverse solute flux, and requires no external pressure. And after the solvent transport from feed side to draw solution side, the diluted draw solution can be regenerated by many other separation methods such as evaporation or distillation [10].

OSFO faces two great challenges: how to enhance the solvent flux in the process and how to improve the solvent resistance of the membrane. For the former, Chung’s group [11] utilized an external hydraulic pressure to enhance the solvent transport through the membrane and proved that organic solvent pressure assisted osmosis (OSPAO) can effectively recycle organic solvents from pharmaceutical products. It is very important to develop FO membrane with good solvent resistance. It means that the OSFO membrane should have strong resistance and long-term stability for a certain organic solvent [12]. However, most of the current commercial FO membranes are mainly developed for water treatment. They are easy to swell when applied in the organic system. This limits its application in the field of organic solvent recovery [13,14].

At present, polyamide TFC membrane is a kind of widely used commercial forward osmosis membrane [13]. A TFC membrane usually consists of a top selective thin layer and a porous substrate [15]. The TFC membrane prepared by interfacial polymerization (IP) can be optimized and regulated by each layer, therefore the FO membrane prepared by this method combines the advantages of porous support layer and dense separation layer, showing high-water flux, excellent salt rejection and good mechanical strength [16]. The porous sub-layer usually is an ultrafiltration or microfiltration membrane like Poysulfone (PS), Polyethersulfone (PES) or Polyvinylidene fluoride (PVDF) membrane and serves as support. However, these substrates fail to maintain their physical integrity in organic solvents because of their tendency to swell or dissolve [17]. Polyimides (PI) have excellent thermal stabilities and good mechanical properties, and they were widely used in photoresists, gas separation membranes, electroluminescent devices, and electrochromic materials [18]. Moreover, PI exhibit satisfactory solvent resistance. Therefore, various types of PI were used as membranes for organic solvent recovery, such as P84 [19,20], Matrimid 5218 [21,22], Ultem 1000 [23]. In this work, PI was used as the substrate to construct the composite forward osmosis membrane for its good solvent resistance.

Introducing an interlayer between the porous substrate and the selective layer, has been proved to be an effective way to regulate the interfacial polymerization and enhance the membrane performance because it can improve reaction interface, increase storage of amine and control amine release [24,25]. Various nanomaterials and organic coatings, such as carbon nanotubes [26], graphene oxide [27] and polyphenol coating [28], were employed to construct interlayers in TFC membrane preparation. Cyclodextrins (CDs) are a class of oligosaccharides consisting of six to eight glucose subunits linked through α-1,4-glycoside bonding [29,30]. These molecules have a high ability to combine a wide range of, especially hydrophobic molecules, due to a hydrophobic cavity and a hydrophilic outer surface [31]. Since CDs can form self-assembled supramolecular structures with other guest molecules through non-covalent bond forces, such as hydrogen bonding, coordination interaction and electrostatic interaction, supramolecular polymers based on the derivatives of CDs have developed rapidly [32]. In 2020, Wu et al. [33] adopted “deep eutectic solvents” to realize a new strategy for the development of solvent-free supramolecular polymers in bulk [34]. In his work, cyclodextrins were used as building blocks in the preparation of deep eutectic supramolecular polymers (DESPs) with various natural acids, wherein complexation was achieved by hydrogen-bond formation between -OH groups of the cyclodextrin and -COOH groups of the natural acid. The DESPs possess excellent processing properties, high viscosity, low fluidity, and good stability in organic solvents, which make them suitable as an interlayer material for solvent-resistant composite membranes.

Monascorubrin is a kind of natural red colorant for many foods such as meats, tofu and wines. Compared with other natural pigments, Monascorubrin has the advantages of easy coloring, low price, good stability, high safety and bacteriostasis. It also has the beneficial effects to health with antioxidant and anti-hyperlipidemia properties [35]. Its production has mainly relied on extraction from fermentation cultures [36]. In the process of extraction and further purification of Monascorubrin, a lot of organic solvent such as ethanol would be used [37]. So, it is of great significance to reduce the cost of organic solvent and recycle organic solvent efficiently in the production of Monascorubrin.

**Figure 1:**
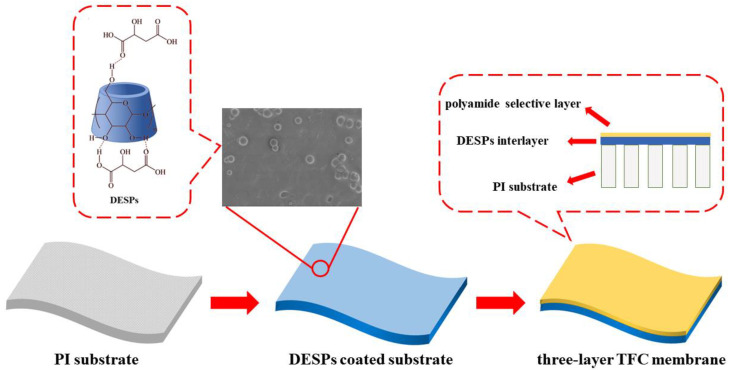
Schematic diagram for the fabrication process and the structures of TFC membranes with deep eutectic supramolecular polymers (DESPs) interlayer.

In this work, we studied the application of FO process in the organic solvent recovery with a novel thin film composite membrane (schematically shown in Figure 1). We prepared the polyimide porous membrane first. Then, the DESPs (formed with CDs and malic acid) with good solvent resistance were coated on the PI substrate, forming the interlayer of the TFC membrane. The thin film solvent-resistant composite membrane was derived after polyamide active layer was formed through interfacial polymerization on the interlayer. Then, we investigated the FO performance of the derived TFC membrane for the separation of Monascorubrin and ethanol.

## 2. Materials and Methods

### 2.1. Materials and Chemicals

Commercial poly(vinylidene fluoride) (PVDF) microfiltration membranes with a nominal pore size of 0.1 μm were provided by Cobetter Filtration Equipment Co., Ltd. (Hangzhou, China). Polyimide (PI) was supplied by the Institute of Process Engineering, Chinese Academy of Sciences (Beijing, China). ß-Cyclodextrin (CD), L-malic acid (MA), and lithium chloride (LiCl) were obtained from Aladdin Reagent Co. Ltd. (Shanghai, China). N-Methylpyrrolidone (NMP), Polyethylene glycol (PEG), 1,6-Diaminohexane, and ethanol were purchased from Sinopharm Chemical Reagent Co., Ltd. (Shanghai, China). Monascorubrin was obtained from Kelong Biotech. Co., Ltd. (Jiangmeng, China). 1,3-phenylenediamine (MPD, 99%) and trimesoyl chloride (TMC, 98%) obtained from Aladdin were used as monomers in the interfacial polymerization process. Deionized (DI) water was supplied by Shanghai Hongkou Baoxing Deionized Water Plant.

### 2.2. Thin-Film Composite Membrane Fabrication

#### 2.2.1. Preparation of PI Substrate

The PI support layer was prepared by the non-solvent-induced phase separation method. A prepared dope solution of 18/16/66 wt% PI/PEG400/NMP (PEG400 as porogen) was cast onto a pre-cleaned glass plate with a glass casting knife and followed by being immersed into a water coagulation bath at room temperature for phase inversion. Solvent NMP exchanged with DI water immediately, then the PI membrane was gradually formed. The as-fabricated membranes were immersed in 5 wt% 1,6-Hexanediamine/isopropanol (1,6-Hexanediamine as crosslinker) solution for 24 h and became cross-linked. Then, the membrane was stored in DI water before use.

#### 2.2.2. Preparation of Triple-Layer TFC Membranes

A certain mass of cyclodextrin and malic acid (molar ratio 1:5 or 1:10) were mixed with deionized water. Then, the mixture was stirred at 80 °C for 3 h. After that, the mixture was heated to 120 °C to evaporate the water. Then, transparent DESPs could be obtained. With the application of spin coating method, cyclodextrin supramolecular polymers with different mole ratios were dropped onto the surface of the PI substrate at a low rotational speed, and the uniform coating of DESPs was derived at a high rotational speed. The membranes with DESPs coating were dried at room temperature. To obtain the polyamide active layer, the PI substrate coated with DESPs interlayer was first immersed in 0.2 wt% MPD/water solution for 3 min. Then, 0.15 wt% TMC/hexane solution was poured onto the top surface of the membrane for 1 min. The modified TFC membrane was washed with isopropanol and then kept in an oven at 60 °C for 8 min. Finally, the obtained membranes were stored in ethanol at least 24 h before use. These modified TFC membranes were denoted as TFC/DESPsn-PI, where n refers to the molar ratio of malic acid to cyclodextrin. The TFC/PI membrane was obtained by interfacial polymerization reacting directly on the PI layer.

### 2.3. Membrane Characterization

The swelling degree (*SD*) of membranes can be calculated after weighing the mass of the membrane before and after soaked in a certain solvent (Equation (1)),

(1) $$SD=\frac{W_{s}-W_{d}}{W_{d}}\times 100$$

where *W*_*s*_ (g) represents the mass of the membranes soaked in different solvents like ethanol, isopropanol (IPA) and ethyl acetate (EAC) for 2 h, respectively, and *W_d_* (g) is the mass of dry membranes.

The porosity (*ε*) of membranes could be calculated with the application of Equation (2):

(2) $$\varepsilon =\frac{m_{1}-m_{2}}{A_{m}l\rho_{w}}$$

where *m*_1_ (g) is the wet weight of the membranes after being fully equilibrated in deionized water, and *m*_2_ (g) is the dry weight of the membranes after drying in the oven at 60 °C for 24 h. *A_m_* (m^2^) is the effective membrane area, *l* (m) is the membrane thickness and *ρ_w_* (g·cm^−3^) is the density of water.

The scanning electron microscope (SEM, S-4800, Hitachi, Tokyo, Japan) was used to investigate the morphological structure of the PI, TFC-PI, and TFC/DESPsX-PI membranes. All the samples were dried and then sputtered with gold before characterization. The atomic force microscope (AFM, Bruker Dimension Icon, Santa Barbara, CA, USA) was used to characterize the roughness of the membrane surface.

The chemical groups of the DESPs-coated substrate and triple-layer TFC forward osmosis membrane surface were analyzed by an attenuated total reflectance Fourier transform infrared spectrophotometer (FTIR, NEXUS FT-IR spectrometer, Themo Nicolet, Madison, WI, USA) with the wavelength range of 500–4000 cm^−1^ and resolution of 2 cm^−1^.

The ethanol contact angle (*CA*) of PI, TFC-PI, and TFC/DESPsX-PI membranes was measured by a contact angle test instrument (JC2000D1, Zhongchen Digital Technology Apparatus Co., Ltd., Shanghai, China) following the sessile drop method at room temperature. To minimize the experiment error, at least three random locations of each membrane were tested to obtain the mean value of the contact angle.

### 2.4. OSFO Membrane Performance Evaluation

The OSFO experimental setup with a lab-scale cross-flow unit was used to evaluate the permeability and selectivity of the prepared triple-layer TFC membranes. The cross-flow membrane cell was designed to have an effective area of 4 × 10^−4^ m^2^. In total, 500 mg/L of Monascorubrin/ethanol solution and LiCl/ethanol solution with different concentrations (0.5 M, 1 M, and 1.5 M) were used as feed solution and draw solution, respectively. The feed and draw solutions were circulated with peristaltic pumps (BT00-600M, KE JIAN, Changzhou, China) at a flow rate of 200 mL/min. The temperatures of the feed and draw solutions were maintained at 30 °C using a heater (NM-50, NOMO, Jiaxing, China). The tests were carried out in two different operation modes, i.e., AL-FS mode (active layer facing the feed solution) and AL-DS mode (active layer facing the draw solution). To determine the solvent flux of membrane, a digital balance (AND EK-3000i, Tokyo, Japan) connected to a computer was utilized to measure and record the weight of the instant feed solution every 20 s.

Since the typical absorption peak of monascorubrin locates at 510 nm, the concentration of monascorubrin in solution can be measured by an ultraviolet-visible (UV-Vis) spectrophotometer. Then, the rejection rate of monascorubrin (*R_f_*) can be calculated by the following Equation:

(3) $$R_{f}=\left(1-\frac{C_{D,F}}{C_{F,F}}\right)\times 100\%$$

where *C_D_*_,*F*_ (mg·L^−1^) and *C_F_*_,*F*_ (mg·L^−1^) are the monascorubrin concentration of the draw solution and feed solution at the end of FO test, respectively.

The solvent flux (*J_w_*, L m^−2^ h^−1^ abbreviated as LMH) and reverse solute flux are determined by Equations (4) and (5), respectively:

(4) $$J_{w}=\frac{\Delta V}{Am\cdot \Delta t}=\frac{\Delta m}{\rho \cdot Am\cdot \Delta t}$$

(5) $$J_{s}=\frac{C_{t}V_{t}-C_{0}V_{0}}{Am\cdot \Delta t}$$

where Δ*V* is the volume change of feed solution, Δ*m* (g) is the weight change of feed solution at regular time intervals Δ*t* (h), *ρ* (g/mL) is the density of feed solution, and *A_m_* (m^2^) is the effective membrane area. *C_t_* and *C*_0_ are the concentration of LiCl (g/cm^3^) of the feed solution at the beginning and end of the FO experiment. *V_t_* and *V*_0_ represent the volume of the feed solution at the beginning and end (L).

In addition, a 600 min OSFO test was performed to evaluate the operation stability of the solvent-resistant composite membrane.

## 3. Results and Discussion

### 3.1. Membrane Characterization

#### 3.1.1. FTIR Spectra

**Figure 2:**
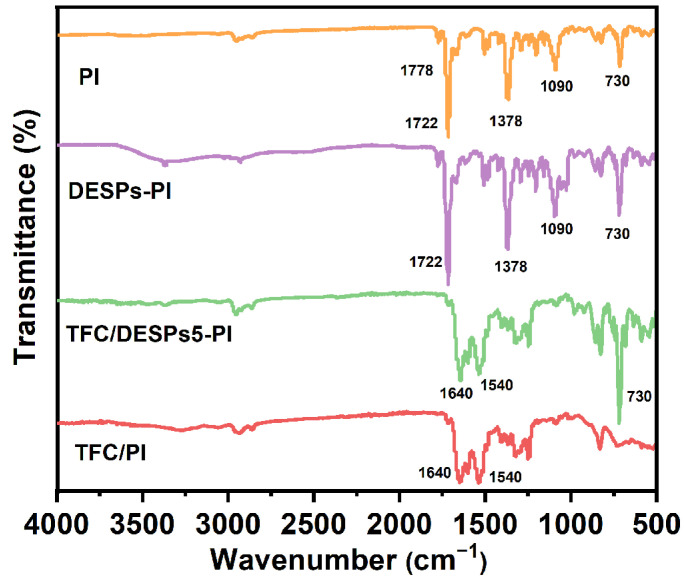
ATR-FTIR spectra of PI, TFC/PI, DESPs-PI, and TFC/DESPs-PI.

Chemical changes in the surface of different membranes after modification were investigated by FTIR. As shown in Figure 2, the PI substrate has two characteristic peaks at 1722 cm^−1^ and 1778 cm^−1^, attributed to the symmetric stretching vibration and the asymmetric stretching vibration of C=O in the imide group, respectively. The absorption peak at 1378 cm^−1^ was attributed to the C-N stretching vibration of the imide group, and the peak at 730 cm^−1^ was caused by the C=O bending vibration in the imide. After coating the interlayer of cyclodextrin supramolecular polymer on the surface of PI substrate, the -OH stretching vibration peak of DESPs appeared at 3400 cm^−1^, and the stretching vibration peak of -CH_2_ in β-cyclodextrin was at 2920 cm^−1^. There was a complex peak at about 1716 cm^−1^, attributed to the C=O characteristic peak of amide and *L*-malic acid. The overlap of C-C/C-O stretching vibration peak of β-cyclodextrin appeared at 1030 cm^−1^. These characteristic peaks indicated that DESPs had been successfully coated on the surface of the PI substrate. Compared with the pristine PI substrate, the TFC membranes with or without interlayer that were obtained after interfacial polymerization both showed the C=O stretching vibration peak of amide at 1640 cm^−1^, and the N-H deformation vibration peak of amide at 1540 cm^−1^, which meant that interfacial polymerization took place on the substrate, and the polyamide active layer formed.

#### 3.1.2. Morphology Characterizations

**Figure 3:**
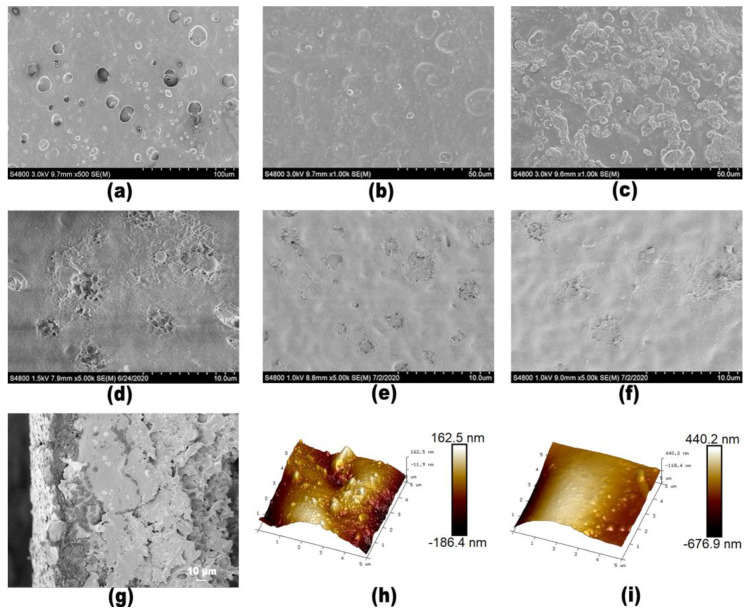
Surface morphologies of (**a**) PI, (**b**) DESPs5-PI, (**c**) DESPs10-PI, (**d**) TFC/PI, (**e**) TFC/DESPs5-PI, (**f**) TFC/DESPs10-PI, (**g**) cross section of TFC/DESPs5-PI, (**h**) AFM of TFC/DESPs5-PI, Ra = 42 nm (**i**) AFM of TFC/DESPs10-PI, Ra = 129 nm.

The surface morphologies of the substrate, the substrate coated with the interlayer, and the triple-layer TFC membranes were observed by SEM. The results are shown in Figure 3. The surface of the PI membrane prepared by the phase inversion method has pores. β-cyclodextrin has a hydrophilic inner wall and hydrophobic cavity, which can self-assemble with small-molecule organic acid through hydrogen bonding to form a supramolecular micelle structure [33], so there are some dispersed spherical micelles on the surface of the substrate coated with DESPs interlayer (Figure 3b). And, there are more spherical micelles on the surface of membrane coated with the DESPs10 interlayer than that on the DESPs5 interlayer (Figure 3c). The reason for this is that more malic acid could cross-link with β-cyclodextrin and formed more supramolecular polymer micelles. On the active layer surface of the two layer TFC membrane, the small “petals” were formed by polyamide scattered on the surface (Figure 3d). However, since abundant hydroxyls of β-cyclodextrin can be reacted with acyl chloride, the surface of the triple-layer TFC membranes, after introduction of the DESPs interlayer, changed to be smoother and denser than the TFC/PI membrane. (Figure 3e,f). Figure 3g shows the cross section of the TFC/DESPs5-PI membrane. The thickness of the selective layer was less than 1μm. And the interlayer was about 20 μm thick. Figure 3h,i were the AFM images of TFC/DESPs5-PI (Ra = 42 nm) and TFC/DESPs10-PI (Ra = 129 nm), which showed that TFC/DESPs5-PI had a smoother surface than TFC/DESPs10-PI. The reason might be that the more viscous DESPa10 made the interlayer uniformity poor, thus the roughness of the surface layer increased.

#### 3.1.3. Solvent Philicity Characterizations

**Figure 4:**
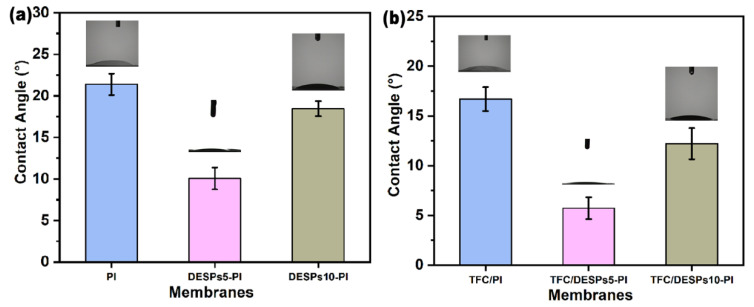
Ethanol contact angles of (**a**) PI, DESPs5-PI, DESPs10-PI substrates and (**b**) TFC/PI, TFC/DESPs5-PI, TFC/DESPs10-PI membranes.

The flux of the organic solvent composite forward osmosis membranes was affected by its solvent philicity, so the ethanol contact angle (ECA) of the membranes was measured. Figure 4a shows that after DESPs were coated on the surface of PI substrates, the ECA decreased. When the molar ratio of β-cyclodextrin to *L*-malic acid was 1:5, the contact angle was less than that of molar ratio 1:10. The reason is that the viscosity of deep eutectic solvent is related to its composition ratio [34]. With the increase of malic acid proportion, the viscosity of the DESPs system increases, which enhances the surface tension of the interlayer and slows down the infiltration of ethanol molecules on the membrane surface. Comparing Figure 4b with Figure 4a, the contact angle of the corresponding composite membrane decreased after interfacial polymerization, owning to a hydrogen bond formed between the polyamide and the hydroxyl group of ethanol, which made ethanol easier to spread on the membrane surface.

#### 3.1.4. The Swelling Degree and Porosity of Membranes

As illustrated in Table 1, after the DESPs5 interlayer was constructed on the PI substrate, the porosity increased to 79.0%. The reason was that a hydrogen bond network in DESPs5 interlayer was formed, which was favorable for the transport of ethanol. The coating of DESPs10 made the interlayer denser and more viscous, and the porosity decreased to 77.3%. It can be found that the porosity of the composite membrane decreases after the polyamide selective layer was obtained by interfacial polymerization, which can be ascribed to the compact structure of the polyamide layer on the surface.

**Figure 5:**
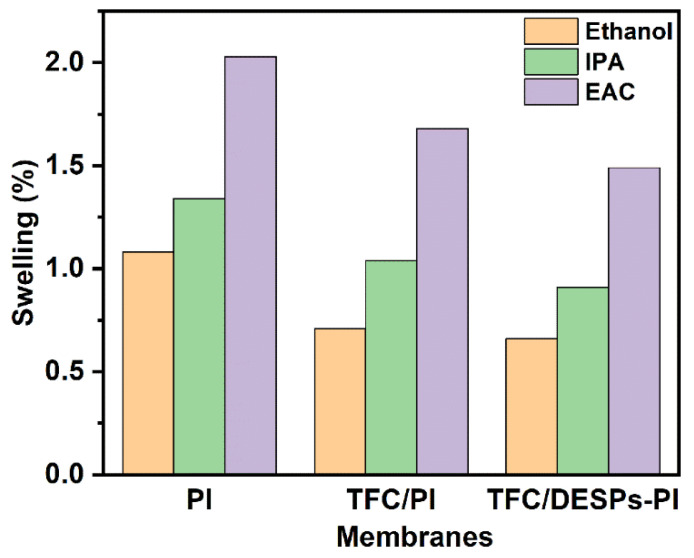
The swelling degree of substrates and TFC membranes in different solvents.

The swelling degree of the PI, TFC/PI and TFC/DESPs5-PI membrane in three different solvents (ethanol, isopropanol and ethyl acetate) were tested. The results are displayed in Figure 5. Since the polarity order of the three solvents is ethanol > isopropanol (IPA) > ethyl acetate (EA), the swelling degree of PI substrate and TFC membranes in ethyl acetate were larger than in ethanol, and the low polarity solvent molecule is more likely to cause the movement of the polymer chain segment. Additionally, the swelling degree of the TFC/PI composite membrane in three different organic solvents was lower than that of the PI substrate. The reason might be that the dense polyamide layer, formed with interfacial polymerization, increases the crosslinking of the composite membrane, weakening the influence of solvents on the polymer chain segment, thus the solvent resistance of the TFC/PI membrane is enhanced. Apparently, after DESPs were introduced into the composite membrane, the swelling degree decreases further. The reason might be that the hydrogen bond network of DESPs creates a higher viscosity, and the structure of the hydrogen bond network is stable in organic solvents [33]. The DESPS interlayer combined with the substrate and polyamide active layer closely and enhanced the stability of the triple-layer composite membrane in organic solvents.

### 3.2. OSFO Membrane Performance Evaluation

Inorganic salts have small molecular weight and high solubility in organic solvents and can produce high-osmotic pressure. Meanwhile, it can stably exist in organic solvents, and would not damage the membrane material. Therefore, at present, inorganic salts are the most common draw solution (DS) used in FO. Since lithium chloride (LiCl) has a small molecular weight and a high solubility in ethanol, Chung’s group [10] adopted LiCl as the DS solute in OSFO and obtained excellent results, proving that LiCl-EtOH can provide a higher driving force for the process of OSFO. Therefore, LiCl-EtOH is utilized as the DS in this study to test the OSFO performance of various composite membranes.

#### 3.2.1. Effect of DESPs Interlayer on the Performance

**Figure 6:**
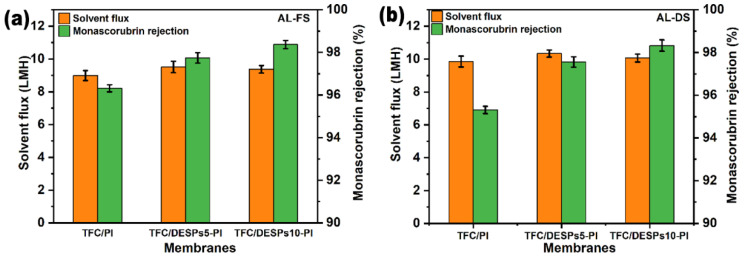
Solvent flux (*J*_*w*_) and monascorubrin rejection (RM) of the TFC/PI, TFC/DESPs5-PI, and TFC/DESPs10-PI membranes. ((**a**). AL-FS mode, (**b**). AL-DS mode, 1.0 M LiCl/ethanol solution as draw solution).

It can be seen from Figure 6 that the solvent flux and the monascorubrin rejection rate of the composite membrane with interlayer increased to some extent. In AL-FS, the solvent flux of TFC-PI membrane was 8.99 LMH. For TFC/DESPs5-PI membrane, the solvent flux increased to 9.51 LMH. The improved solvent flux was consistent with the results of the membrane solvent philicity in the contact angle test. And the following factors could explain the improved monascorubrin rejection. Firstly, the introduction of DESPs5 brings a large amount of hydrogen bonding, and the β-cyclodextrin forming the interlayer has a cavity structure to prevent monascorubrin from penetrating through the composite membrane. Secondly, due to the larger solvation size of monascorubrin in ethanol, it is more difficult to penetrate through the three-layer structure. In AL-FS mode, the monascorubrin rejection rate was 96.8% for the pristine TFC/PI membrane, while for the TFC DESPs5-PI membrane, it increased to 98.4%.

Meanwhile, the molar ratios of β-cyclodextrin to L-malic acid also had influence on the solvent flux and the monascorubrin rejection. In both operating modes, the flux of TFC/DESPs10-PI was slightly lower than that of TFC/DESPs5-PI, while the rejection rate increased. The probable reason was that the increase of malic acid leads to the formation of a more compact hydrogen bond network structure, and the viscosity of DESPs increases, which was not conducive to the infiltration of ethanol molecules. At the same time, the monascorubrin retention effect was improved. This proves that constructing the DESPs interlayer is an effective way to improve the performance of solvent-resistant forward osmosis membranes.

The reverse solute flux data are listed in Table 2. For the TFC/PI, TFC/DESPs5-PI and TFC/DESPs10-PI membrane, their low reverse solute flux showed that the membranes had a stronger rejection for LiCl. Among them, the TFC/DESPs5-PI membrane had the lowest reverse solute flux and *J*_*s*_/*J*_*w*_ value, meaning that TFC/DESPs5-PI showed the best solvent FO performance.

#### 3.2.2. Effect of Draw Solution Concentration on the Performance

**Figure 7:**
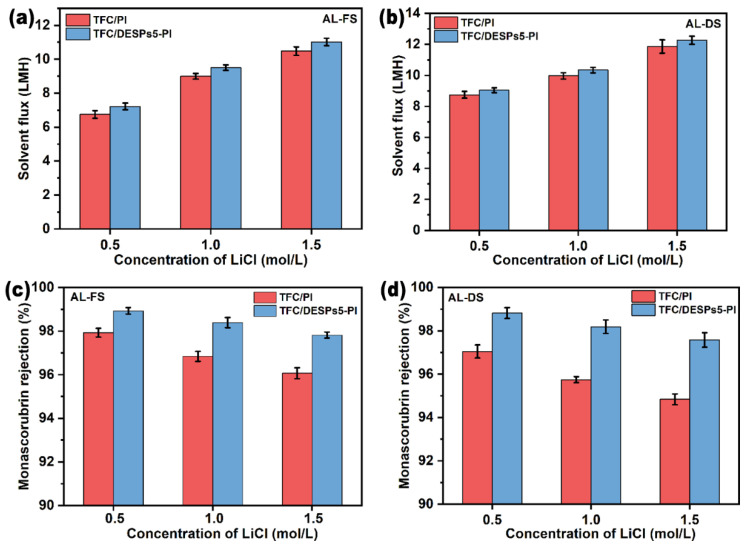
Solvent flux (*J*_*w*_) (**a**,**b**) and monascorubrin rejection (RM) (**c**,**d**) of the TFC/PI and TFC/DESPs5-PI membranes with different concentrations of draw solution. ((**a**,**c**) AL-FS mode, (**b**,**d**) AL-DS mode).

The driving force of FO is the osmotic pressure difference between the draw solution and the feed solution. Hence, the concentration of draw solution will influence the performance of FO membrane. As shown in Figure 7a,b, the solvent flux improved gradually with the increase of draw solution concentration, but the increasing trend decreased, attributed to the occurrence of internal concentration polarization (ICP) in the porous support layer of the composite membrane [38]. For instance, the osmotic pressure of 0.5 M LiCl/ethanol solution (draw solution) is about 16.73 bar. In Al-FS mode, the flux of TFC/DESPs5-PI is about 7.22 LMH. However, the flux is 11.02 LMH in the same operation mode with 1.5 M LiCl/ethanol draw solution, whose osmotic pressure is about 41.14 bar. The permeation flux did not show a linear increase trend with the increase of the concentration of the draw solution. On the one hand, the increase in the concentration of the draw solution generated a greater driving force. On the other hand, a higher concentration of draw solution leaded to more serious ICP of the composite membrane and reduced the effective osmotic pressure difference, thus affecting the growth of permeation flux and resulting in a lower flux than the theoretical value.

Figure 7c,d reflected the relationship between the monascorubrin rejection rate and the concentration of the draw solution during the organic solvent forward osmosis process. As the concentration of the draw solution increased, the monascorubrin rejection rate of the FO composite membrane decreased. The possible reason is as follows: with the increase of osmotic pressure of the draw solution, the driving force of organic solvent FO increased, and the ethanol flux increased accordingly. The increase of permeation flux would also accelerate the rate of monascorubrin through the membrane. Therefore, the retention rate of monascorubrin decreased.

Comparing the FO performance in different operation mode (AL-DS mode or AL-FS mode), for the same membrane, the solvent flux in AL-DS mode was higher than that in AL-FS mode, while the monascorubrin rejection rate in AL-DS mode was lower than that in the AL-FS mode. The reason was that the effective osmotic pressure difference between the two sides of the membrane in AL-FS mode (dilutive ICP occurred) is smaller than that in AL-DS mode (concentrative ICP occurred). Further, in AL-DS mode, LiCl in draw solution would enter the support layer of the composite membrane due to reverse diffusion, thus increasing the resistance of ethanol to permeate the membrane.

### 3.3. OSFO Membrane Stability Evaluation

**Figure 8:**
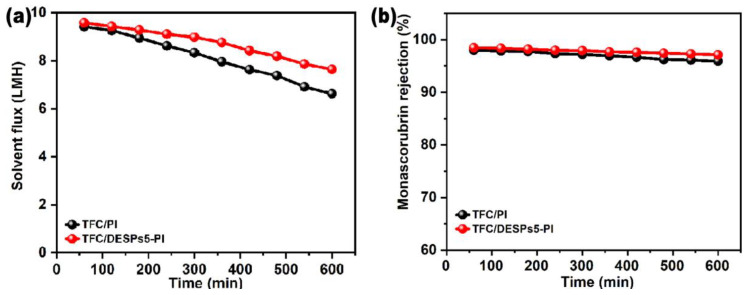
Stability test of (**a**) solvent flux and (**b**) monascorubrin rejection over time by organic solvent-resistant forward osmosis membranes with and without interlayer (AL-FS mode, 1.0 M LiCl/ethanol solution as draw solution).

To evaluate the stability of solvent-resistant composite membranes, solvent flux and monascorubrin rejection rate were tested for 600 min with the TFC/DESPs5-PI membrane in contrast to the TFC/PI membrane. It was shown that, in Figure 8, the flux and rejection rate of the two membranes decreased with time. On the one hand, ethanol in the feed solution diffused to the draw solution side, which decreased the concentration of draw solution and resulted in a decreased driving force of FO. On the other hand, with time prolonging, the diffusion of solution in two sides of the membrane might lead to part of membrane pores’ blocking and fouling. In addition, the composite membranes in an organic solvent for a long time would swell and destroy the membrane structure to a certain degree, reducing the separation performance of the composite membrane. However, comparing the performance of the TFC/DESPs5-PI membrane with the TFC/PI membrane, the attenuation of the TFC/DESPs5-PI membrane was smaller than that of the TFC/PI membrane, indicating that the stability of the membrane with DESPs interlayer was improved.

## 4. Conclusions

In this study, DESPs were employed to construct the interlayer on the PI substrate by a coating method with cyclodextrin and L-malic acid, and the triple-layer composite membrane was derived after interfacial polymerization on the interlayer. The triple-layer membrane exhibited improved performance and good stability in the forward osmosis of organic solvent. FTIR and SEM characterization results confirmed the effective introduction of the DESPs interlayer. In comparison with the pristine TFC-PI membranes, the porosity and swelling degree of the composite membrane with the DESPs interlayer reduced, and the organic solvent philicity increased. Accordingly, the composite membrane with the interlayer showed higher solvent flux and a better rejection rate. In addition, TFC/DESPs5-PI presented good stability in a 600 min FO operation. The operating conditions also had influences on the OSFO performance. The solvent flux increased with the concentration of the draw solution, while the monascorubrin retention rate decreased.

## 5. Patents

There is a Chinese invention patent (CN202011243663.8) resulting from the work reported in this manuscript.

## Figures and Tables

**Table 1:** Porosity of substrates, substrates with interlayer and TFC membranes.

Substrates/ Membranes	PI	DESPs5-PI	DESPs10-PI	TFC/PI	TFC/ DESPs5-PI	TFC/ DESPs10-PI
Porosity (%)	76.1	79.0	77.3	45.4	48.6	47.0

**Table 2:** Solvent flux *J_w_*, reverse solute flux *J_s_*, and *J_s_*/*J_w_* of the membranes.

Membrane	Solvent Flux (*J_w_*, LMH)	Reverse Solute Flux (*J_s_*, gMH)	*J_s_*/*J_w_* (g/L)
TFC/PI	8.99	3.16	0.352
TFC/DESPs5-PI	9.51	2.28	0.240
TFC/DESPs10-PI	9.37	2.74	0.292

1.0 M LiCl as draw solution, AL-FS mode.

## Data Availability

The data presented in this study are available on request from the corresponding author.

## References

[1] Benvenutti L., Zielinski A.A.F., Ferreira S.R.S. (2019). Which is the best food emerging solvent: IL, DES Or NADES?. Trends Food Sci. Technol..

[2] Farrán A., Cai C., Sandoval M., Xu Y.M., Liu J., Hernáiz M.J., Linhardt R.J. (2015). Green solvents in carbohydrate chemistry: From raw materials to fine chemicals. Chem. Rev..

[3] Jiménez-González C., Poechlauer P., Broxterman Q.B., Yang B.S., Ende D.A., Baird J., Bertsch C., Hannah R.E., Dell Orco P., Noorman H. (2011). Key green engineering research areas for sustainable manufacturing: A perspective from pharmaceutical and fine chemicals manufacturers. Org. Process Res. Dev..

[4] Aboagye E.A., Chea J.D., Yenkie K.M. (2021). Systems level roadmap for solvent recovery and reuse in industries. iScience.

[5] Furukawa H., Cordova K.E., Keeffe M., Yaghi O.M. (2013). The Chemistry and applications of metal-organic frameworks. Science.

[6] Muralikrishna I.V., Manickam V. (2017). Hazardous waste management. Environ. Manag..

[7] Shirsat S.P. (2013). Separation of isobutyl alcohol and isobutyl acetate by extractive distillation and pressure-swing distillation: Simulation and optimization. Sep. Purif. Technol..

[8] Ruthusree S., Sundarrajan S., Ramakrishna S. (2019). Progress and perspectives on ceramic membranes for solvent recovery. Membranes.

[9] Lively R.P., Sholl D.S. (2017). From water to organics in membrane separations. Nat. Mater..

[10] Cui Y., Chung T.S. (2018). Pharmaceutical concentration using organic solvent forward osmosis for solvent recovery. Nat. Commun..

[11] Cui Y., Chung T.S. (2019). Solvent recovery via organic solvent pressure assisted osmosis. Ind. Eng. Chem. Res..

[12] Marchetti P., Jimenez Solomon M.F., Szekely G., Livingston A.G. (2014). Molecular separation with organic solvent nanofiltration: A critical review. Chem. Rev..

[13] Zhao S.F., Zou L., Tang C.Y.Y., Mulcahy D. (2012). Recent developments in forward osmosis: Opportunities and challenges. J. Membr. Sci..

[14] Chung T.S., Li X., Ong R.C., Ge Q.C., Wang H.L., Han G. (2012). Emerging forward osmosis (FO) technologies and challenges ahead for clean water and clean energy applications. Curr. Opin. Chem. Eng..

[15] Cath T.Y., Childress A.E., Elimelech M. (2006). Forward osmosis: Principles, applications, and recent developments. J. Membr. Sci..

[16] Yasukawa M., Mishima S., Tanaka Y., Takahashi T., Matsuyama H. (2017). Thin-film composite forward osmosis membrane with high water flux and high pressure resistance using a thicker void-free polyketone porous support. Desalination.

[17] Peyravi M., Rahimpour A., Mohsen Jahanshahi M. (2012). Thin film composite membranes with modifified polysulfone supports for organic solvent nanofiltration. J. Membr. Sci..

[18] Liaw D.J., Wang K.L., Huang Y.C., Lee K.R., Lai J.Y., Ha C.S. (2011). Advanced polyimide materials: Syntheses, physical properties and applications. Prog. Polym. Sci..

[19] See-Toh Y.H., Silva M., Livingston A. (2008). Controlling molecular weight cut-off curves for highly solvent stable organic solvent nanofiltration (OSN) membranes. J. Membr. Sci..

[20] Soroko I., Livingston A. (2009). Impact of TiO_2_ nanoparticles on morphology and performance of crosslinked polyimide organic solvent nanofiltration (OSN) membranes. J. Memb. Sci..

[21] Guiver M.D., Robertson G.P., Dai Y., Bilodeau F., Kang Y.S., Lee K.J., Jho J.Y., Won J. (2002). Structural characterization and gas-transport properties of brominated matrimid polyimide. J. Polym. Sci. Part A Polym. Chem..

[22] Liu S.L., Chng M.L., Chung T.S., Goto K., Tamai S., Pramoda K.P., Tong Y.J. (2004). Gas-transport properties of indan-containing polyimides. J. Polym. Sci. Part B Polym. Phys..

[23] Kurdi J., Tremblay A.Y. (2001). The influence of casting solution structure on the microporosity of polyetherimide gas separation membranes prepared by the coagulation post-leaching method. J. Membr. Sci..

[24] Yang Z., Sun P.F., Li X.H., Gan B.W., Wang L., Song X.X., Park H.D., Tang C.Y. (2020). A critical review on thin-film nanocomposite membranes with interlayered structure: Mechanisms, recent developments, and environmental applications. Environ. Sci. Technol..

[25] Dai R., Li J., Wang Z. (2020). Constructing interlayer to tailor structure and performance of thin-film composite polyamide membranes: A review. Adv. Colloid Interfac..

[26] Liao M., Zhu Y., Gong G., Qiao L. (2022). Thin-Film Composite Membranes with a Carbon Nanotube Interlayer for Organic Solvent Nanofifiltration. Membranes.

[27] Li Y., Li C., Li S., Su B., Han L., Mandal B. (2019). Graphene oxide (GO)-interlayered thin-film nanocomposite (TFN) membranes with high solvent resistance for organic solvent nanofiltration (OSN). J. Mater. Chem. A.

[28] Zhang X., Lv Y., Yang H., Du Y., Xu Z. (2016). Polyphenol coating as an interlayer for thin-film composite membranes with enhanced nanofiltration performance, ACS Appl. Mater. Interfaces.

[29] Engeldinger E., Armspach D., Matt D. (2003). Capped cyclodextrins. Chem. Rev..

[30] Del Valle E.M. (2004). Cyclodextrins and their Uses: A Review. Process Biochem..

[31] Larsen K.L. (2002). Large cyclodextrins. J. Incl. Phenom. Macro Chem..

[32] Harada A., Takashima Y., Yamaguchi H. (2009). Cyclodextrin-based supramolecular polymers. Chem. Soc. Rev..

[33] Wu S.G., Cai C.Y., Li F.F., Tan Z.J., Dong S.Y. (2020). Deep eutectic supramolecular polymers: Bulk supramolecular materials. Angew. Chem. Int. Ed..

[34] Abbott A.P., Harris R.C., Ryder K.S. (2007). Application of Hole Theory to define ionic liquids by their transport properties. J. Phys. Chem. B.

[35] Vendruscolo F., Bühler R.M.M., de Carvalho J.C., de Oliveira D., Moritz D.E., Schmidell W., Ninow J.L. (2016). Monascus: A reality on the production and application of microbial pigments. Appl. Biochem. Biotech..

[36] Akihisa T., Tokuda H., Ukiya M., Kiyota A., Yasukawa K., Sakamoto N., Kimura Y., Suzuki T., Takayasu J., Nishino H. (2005). Anti-tumor-initiating effects of monascin, an azaphilonoid pigment from the extract of monascus pilosus fermented rice (Red-mold rice). Chem. Biodivers..

[37] Agboyibor C., Kong W.B., Zhang A.M., Niu S.Q. (2019). Nutrition regulation for the production of monascus red and yellow pigment with submerged fermentation by monascus purpureus. Biocatal. Agric. Biotechnol..

[38] Gray G.T., McCutcheon J.R., Elimelech M. (2006). Internal concentration polarization in forward osmosis: Role of membrane orientation. Desalination.

